# Interest and Confidence in Death Education and Palliative Psychology in Italian and Indian University Students of Psychology: Similarities and Differences

**DOI:** 10.3390/bs13020183

**Published:** 2023-02-17

**Authors:** Gianmarco Biancalani, Rekha Wagani, Lucia Ronconi, Matteo Cornacchini, Ines Testoni

**Affiliations:** 1 Department of Philosophy, Sociology, Education and Applied Psychology, University of Padova, 35131 Padova, Italy; 2 Amity Institute of Behavioral and Allied Sciences, Amity University Mumbai, Mumbai 410206, India; 3 IT and Statistical Services, Multifunctional Pole of Psychology, University of Padova, 35131 Padova, Italy

**Keywords:** death education, palliative psychology, university students, interest in the end-of-life, confidence in the end-of-life

## Abstract

Teaching death education and palliative psychology in universities has proven to be of great importance, especially in the health professions. The present study aims to evaluate the similarities and differences in interest and confidence in death education and palliative psychology between university psychology students from two culturally different countries: Italy and India. For this study, 63 Italian and 35 Indian psychology students were recruited to take part in a course on death education and palliative psychology. The results showed the positive impact of a death education and palliative psychology course on the training of professionals. In particular, this course was useful in helping students become familiar with and learn how to manage future professional situations related to death and dying. Specific differences between the two countries also emerged, particularly with regard to their approach to the end-of-life field, due to different cultural contexts. There is still much to be done by institutions to improve the dissemination and academic teaching of this area, which in turn can promote job opportunities for young people and encourage them to work in this field.

## 1. Introduction

The origins of death education can be strictly linked to the death awareness movement during the 1960s and the 1970s, which tried to help people overcome death anxiety through cognitive resources [1]. The knowledge that death is inevitable comes up both when we experience death and when we talk about it [2]. In this context, death education aims to reduce death anxiety by acquiring knowledge and improving communication about death and how to overcome grief and loss, thereby reducing pain and distress for those involved [3,4].

Palliative psychology is a field that attempts to alleviate psychological and existential distress and pain both in patients with severe and advanced diseases as well as in their families and caregivers [5]. Through a holistic approach, palliative psychology aims to address the psychological and existential needs in the different phases of terminal illness, including the end-of-life and grieving process, with attention to the patient and family members, as well as to the palliative care team, having at its disposal suitable tools for the diagnosis and treatment of complex clinical pictures, which the end of life entails [6]. Healthcare professionals in various disciplines are often unprepared to work with dying or bereaved patients [7], whilst palliative psychologists have the skills to meet the psychological needs of patients, detect the impact of the disease on their families and assess and manage the resulting distress [6]. Confusion, fear, existential questions, and painful feelings related to one’s losses often emerge when helping people who are facing a terminal illness and, if repeated over time, can lead to burnout [8]. This is why death education courses are essential in the university training of these professionals [9,10]. 

Since the emergence of the death awareness movement, courses on death education and palliative care have started to appear in universities, especially in degree programmes focused on medicine, nursing, and psychology [11,12]. According to some studies, participation in a death education course significantly reduces death anxiety and fear of death in healthcare students [7,8,13]. International studies on the impacts of death education and palliative psychology courses on university students have proven useful in increasing students’ interest in this area, making them feel more comfortable in dealing with the topic of death and in handling the difficulties resulting from such a painful and stressful situation with competence and professionalism [7,8,9,14,15,16]. Unfortunately, theories and practices regarding end-of-life are rarely addressed in study courses, resulting in increased fear of this topic [7,8,9]. University students from different countries have expressed the need for an in-depth study of death education to help them become more confident in dealing with this highly challenging field [9,10,14,15,16,17]. This is in line with the current situation in Italy and India. Regarding Italian university education, students complain of a lack of preparation in death education and palliative psychology and in the management of death anxiety and the relationship with the dying [9,10,17]. Most health profession curricula in India contain very few courses in palliative care, let alone palliative psychology and death education [18]. Inadequate training and education towards these topics are recognised by students from Italy and other countries who would like to explore them further, as they are interested in the end-of-life area for their future work [9]. Less intolerance of life’s ambiguities, a higher age, and having cared for a person until their death contribute to greater confidence in their working abilities [9,19]. According to research, viewing death as a total annihilation leads to a greater level of despair and anxiety than viewing death as passing [20]. The projection of one’s identity beyond death, as considered by terror management theory, influences an individual’s hope, resilience, and ability to cope with stressful situations [21]. Such self-representation allows individuals to maintain the cultural framework through which meaning is given to everyday life [21]. In fact, giving life meaning and conceiving death as a passage are factors that have been closely linked to lower levels of distress, anxiety, and depression [20,22,23,24]. 

Regarding the national context of the two countries, palliative care and assisted suicide are legally allowed in Italy, but euthanasia is an illegal and punishable practice [6]. In India, there is a lack of legislative clarity regarding palliative care that, with assisted suicide and euthanasia, can be considered illegal practices [25,26]. This may be due to the strong impact the religious sphere has on these two countries: 85% of the Italian population belongs to a religious faith, of whom about 80% are Christians [27], whilst in India, there is a greater plurality of religions that have coexisted peacefully for millennia [28]. The most widespread religion is Hinduism (80.5% of the Indian population), but there are some minorities who practice Islam, Christianity, Buddhism, Jainism, Sikhism, and other smaller religious beliefs [29].

The present study builds on a previous international study that investigated the confidence and interest of undergraduate psychology students in death education and bereavement care in five countries [9]. This study aims to investigate similarities and differences regarding interest and confidence in two groups of students from two different countries (Italy and India) who participated in a course on death education and palliative psychology. Therefore, we want to see how students who come from different cultural contexts approach end-of-life topics, the impacts of these topics on the students, and whether they find this type of training useful in their future careers.

## 2. Materials and Methods

### 2.1. The Italian Course

The Italian course consisted of 42 academic hours of teaching, divided into two weekly meetings of two hours each, for a total duration of three months. The contents addressed in the course were as follows: Death and dying: biological and cultural factors and their transformation in history; psychology, sociology and philosophy of religion and death; funerary and symbolic rituals of the passing away; terror management theory and the main theories about death and dying; palliative care and the medical humanities approach; quality of life and pain management in terminality; bioethics and bio-laws about death; social management of dying; advance directives on treatment choice; grieving, complicated bereavement, and traumatic bereavement; and psychological intervention in the field of death and end-of-life. Approximately four hours of frontal lessons were dedicated to each topic. The delivery of the course was structured in the form of lessons, which included moments of personal reflection, group discussions, documentaries, and meetings with national and international experts.

### 2.2. The Indian Course

The Indian course consisted of 60 academic hours of teaching, divided into three weekly meetings of three hours each, for a total duration of five months. The contents were divided into five modules. All modules and contents featured several activities and were divided so that most of the contents received an equal number of hours from the overall average of 15 h. The topics covered in the five modules of the course were as follows: Palliative care: Structures and processes of care; Domains of palliative care; Care of the caregivers and patient at the end of life; Legal and ethical aspects of care; and Palliative care in India. Each module covered four different contents related to the module, covering a total of 20 different topics, including models, stages, physical, psychological, and spiritual domains of palliative care; components of care; end-of-life goals; post-palliative care and process of grief and bereavement; health care ethics; and legal and psychiatric aspects in palliative care. Various modes were used to deliver the course effectively to the participants, including group discussions, personal reflections, movies and documentaries based on end-of-life, discussions of previous legal cases, and presentations, among others.

### 2.3. Participants

The psychology students who were recruited were enrolled in the courses of death education and palliative psychology at the two respective universities located in Italy and India. The research project was presented at the beginning of the course in each class. The inclusion criteria were as follows: students who attended the course and those who participated in the classroom lessons. Research participation was voluntary and anonymous; it did not provide prizes for those who participated or penalties for those who did not participate.

In Italy, 63 students correctly completed the questionnaire before the course, and 51 did so after the course. In India, 25 students answered the questionnaire before the course, and 36 students did the same after the course. 

### 2.4. Data Collection

The quantitative data were collected through a questionnaire survey that lasted about 10 min each. The surveys were completed by the students both before and after the course. A link to access the questionnaire was sent to them by the researchers. At the end of the questionnaire, we provided a space wherein those who wanted to be contacted to answer questions during a subsequent online written interview could leave their email address. Students who left their email addresses were contacted by the researchers, who provided them with the link to answer the questions for the online written interview. Both the questionnaires and the interviews were administered anonymously, allowing the students to express themselves freely in relation to the course they had attended.

The study used a mixed-method approach, as this allowed for a more in-depth investigation of the similarities and differences related to the students’ cultural contexts of origin and their approaches to death education and palliative psychology. The qualitative interviews made it possible for them to expand the topics in the quantitative questionnaire, thus yielding more in-depth results.

### 2.5. Measures

#### 2.5.1. Quantitative

The quantitative online questionnaire was taken from a previous study that investigated the levels of interest and confidence among students in death education and palliative care [9]. It consisted of the following parts:

Demographic questions: these questions aimed to collect data on the participants’ age, gender, marital status, religion, level of observance, year of university, experience as a formal caregiver to end-of-life clients (e.g., at a hospice, hospital, or non-governmental organisation), loss of someone close in the last two years, and whether they had anyone close who had a terminal illness at the time of the survey.

Interest questions: four questions rated on a 5-point Likert scale (1 = very disinterested, 5 = very interested) were asked to investigate students’ interest in the following: the themes of death education, loss, mourning, and palliative care; obtaining practical/clinical competence for working with clients coping with end-of-life conditions, bereavement and/or palliative care; acquiring theoretical knowledge about end-of-life conditions, bereavement and/or palliative care; and working with clients coping with end-of-life conditions, bereavement and/or palliative care. The pre- and post-test Cronbach’s alpha values were 0.69 and 0.86, respectively.

Death representation questions: two questions rated on a 5-point Likert scale ranging from 1 (strongly disagree) to 5 (strongly agree) were asked to investigate students’ representation of death as a passage or as a total annihilation: (1) “Death is terminal, and there is nothing after death” and (2) “Death is a passage to another dimension where existence somehow continues”. 

Intolerance of ambiguity questions: two questions rated on a 5-point Likert scale ranging from 1 (strongly disagree) to 5 (strongly agree) were asked to investigate students’ intolerance of ambiguity in life: (1) ‘The ambiguities in life stress me’ and (2) “Uncertainty makes me uneasy, anxious or stressed”. The pre- and post-test Cronbach’s alpha values were both 0.74.

Confidence questions: Students were asked the extent to which they agreed with five statements [30], which were rated on a 5-point scale ranging from 1 (strongly disagree) to 5 (strongly agree) regarding their confidence in dealing with situations in the field of death and end-of-life. The pre- and post-test Cronbach’s alpha values were 0.93 and 0.90, respectively. Example item: “I am confident about helping people with their bereavement”.

#### 2.5.2. Qualitative

The online interviews, which lasted about 45 min each, consisted of six questions that investigated the following points: (1) the importance of carrying out death education and palliative psychology interventions accompanying death; (2) the emotions aroused by the subject of death in the respondent and the skills in managing the moments in which death is lived or talked about; (3) professional motivation to work in the future as a palliative psychologist; (4) personal meanings given to life, death, and spirituality; (5) how society experiences the theme of death and end of life, and what strategies to implement to improve the dissemination of the theme at a social level; and (6) the impacts of the COVID-19 pandemic on the themes of death, loss, and bereavement management.

### 2.6. Data Analysis

#### 2.6.1. Quantitative

In Italy, 63 out of 67 students who were enrolled in the course (94% response rate) were recruited and responded to the survey before the course, whilst 51 students completed the questionnaire after the course (76% response rate). A total of 35 students (52% response rate) correctly completed the questionnaire both before and after the course. 

In India, 51 students were enrolled in the course, but many students were unable to complete the questionnaire either before or after the course. Thus, only 25 and 36 students answered the questionnaire before and after the course, respectively, resulting in corresponding response rates of 49% and 71%. Given that participation in the study was voluntary and anonymous, the students were free to decide whether they wanted to complete the questionnaire. 

All the quantitative data gathered from the survey were analysed using SPSS software. Descriptive statistics for demographic and experience variables were computed for each country before and after the course. Differences between countries were evaluated by Chi-square test for categorical variables and by t-test for continuous variables. Regression analysis on the target variables was performed to evaluate the contributions of demographic variables, experience variables, and country before and after the course. The course effect was evaluated only for a subsample of Italian students (where it was possible to match the questionnaires completed before and after the course) by t-test and Cohen’s d as a measure of effect size.

#### 2.6.2. Qualitative

Textual data were collected online and analysed using qualitative thematic analysis, a theoretically flexible and well-suited method for studying participants’ experiences and perceptions [31]. This method allows for the recognition of key patterns of meanings and concepts across a given dataset without any preconceptions [31,32]. For the current study, the thematic analysis was conducted according to the six phases outlined by Braun and Clarke [31]: familiarisation with the data, coding, generating initial themes, reviewing themes, defining and naming themes, and writing the report. The approach adopted in the current study is inductive, which involves the process of coding and creating themes from data without preconceptions. The data were first analysed separately, as they were in the corresponding language of each country, and then compared. As strong similarities in coding emerged, it was possible to divide the data into the same themes for both countries.

## 3. Results

### 3.1. Quantitative Results

#### 3.1.1. Country Differences

Several differences between Italian and Indian students were found before and after the course (Table 1). For example, Italian students were younger, mostly women, mostly of Christian religion or without religion. There were even fewer Italian students who subscribed to the Hindu religion. They also tended to have lower levels of religiosity than the Indian students. Furthermore, their experiences about end of life, bereavement, and/or palliative care varied. In particular, Indian students had more knowledge in this field than Italian students because they were mainly in the second year of the master’s degree programme (96% in the Indian sample vs. 25% in the Italian sample).

Regression analysis on the target variables (interest in end-of-life, bereavement and/or palliative care topics; confidence in this field; death’s representations; and the intolerance of ambiguity) was performed to evaluate the contributions of the demographic variables, experience variables, and country before and after the course (Table 2). On the one hand, the results before and after the course were quite different for interest (R-square of 25% before and 18% after) and confidence (R-square of 57% before and 20% after), with a central role of previous knowledge in this field only before the course. In fact, having read something about end-of-life, bereavement and/or palliative care were all positively associated with interest and confidence only before the course (B = 0.38, SE = 0.17, *p* = 0.025 and B = 0.59, SE = 0.25, *p* = 0.022, respectively). Furthermore, membership in the Indian sample was associated with greater confidence only before the course (B = 1.32, SE = 0.32, *p* < 0.001). The results before and after the course, on the other hand, were similar for death’s representation (R-square about 30% both times) and the intolerance of ambiguity (R-square about 10% both times), with a central role of religious level for the first two variables and without significant associations for the last variable. In fact, higher religiosity was associated with a higher view of death’s representation as a passage (B = 0.81, SE = 0.17, *p* < 0.001) and lower death’s representation as terminal (B = −0.79, SE = 0.18, *p* < 0.001). Furthermore, membership in the Indian sample was associated with a greater view of death’s representation as terminal, but only before the course (B = 1.00, SE = 0.50, *p* = 0.049).

#### 3.1.2. Course Effect Evaluation for the Italian Sample

The effects of participation in the course were evaluated only for 35 Italian students, for whom it was possible to match the questionnaires completed before and after the course (Table 3). Overall, students showed a higher level of confidence (t = 4.80, df = 34, *p* < 0.001) and positively modified their concept of death’s representation after the course. In particular, they increased death’s representation as a passage (t = 3.68, df = 34, *p* < 0.001) and decreased death’s representation as terminal (t = −2.95, df = 34, *p* = 0.006). In particular, the course showed a strong effect size for confidence (d = 0.81) and a moderate effect size for death’s representation (d = 0.62 for death as a passage and d = −0.50 for death as terminal). 

### 3.2. Qualitative Results

Four themes emerged from the analysis of the written interviews conducted with both student groups:

- beliefs about life and death;
- social avoidance of the topic of death;
- areas of death education use;
- impact of the death education course.

#### 3.2.1. First Theme: Beliefs about Life and Death

The first theme focused on the participants’ views of life and death. Seven Italian students reported that they were influenced by the religious education they received, as reported, for example, by this student:

I grew up in a Catholic family and had very few doubts about the most important concepts of this faith, even throughout my adolescence (many other concepts always seemed secondary and invented by man, and I don’t particularly care about them).

Furthermore, although these seven students moved away from the religious education they received, they retained the spiritual content of the religious tradition. This sentiment was expressed by another student:

For many years, I was a staunch Christian believer, so I have always conceived life as a gift to be treasured, to be made the best possible from, in a later afterlife perspective. After my distancing from faith, I have not stopped viewing life as an opportunity and have always lived considering myself fortunate with what I have. At the same time, although I no longer believed in a god, I never abandoned the idea that there might be something mystical.

Along similar lines, a total of 26 pupils from India said that religion helped them comprehend what life and death signify. One student, for instance, shared the following:

My views about death and life are strongly connected with religious beliefs. I believe that it is an inevitable process that cannot be skipped. We are born alone, and we have to leave the world alone. We are here to create a connection and to make a storyline. It is up to you to do your deeds.

The participants from both countries perceived life and death as integrated. Regarding considerations about life, three Italian participants saw it as an opportunity to fulfil themselves, as reported by one student: “Life, in my opinion, is a unique opportunity. Every human being has to realise their happiness and achieve their goals”. Another Italian student, who saw life as a journey, shared the following:

I think life is a journey whose destination is known and the same for everyone. A life without passion is like a journey in the fog—you arrive at your destination, and you haven’t even enjoyed the scenery. Maybe it is simple; maybe it is simplistic, but this is my philosophy of life. Accordingly, I try to cultivate some interests and to seek happiness for myself and my loved ones without harming others.

Similar to the above views, Indian students also expressed that the aim of life is to find connections, carry out good deeds and prepare for a better death: “The meaning of life is connecting with the purpose, and for that purpose to guide us into development and service. We have this intrinsic, burning desire to do something or be something”. Furthermore, Indian students perceived that an awareness of one’s mortality makes one a better person, as exemplified by the following answer:

If an individual is conscious that they might or people around them might die at any given point of time, then we will be more forgiving and loving towards others and be better humans ourselves. Nobody wants their last words to someone be about something bad or rude. We would like our last words or actions to be good and kind.

The one common belief that emerged from both countries’ participants concerned our existence in life after death. In this regard, one Italian student expressed this view:

I adduce a Christian–Catholic religious view and find that our souls cannot end but continue after death. Although I am not a particularly religious person, I believe in life after death, similar to what Christianity says. I do not believe in a clear-cut heaven/hell/purgatory division, because I fear and shy away from the thought of eternal damnation. I see life as the antechamber to something more beautiful and greater, as a kind of preparation for…

The same view was reported by another student who said, “I believe, however, in the existence of a further life after death, although I do not know in what form and in what way this life will be”.

Compared with their Italian peers, the Indian students gave more emphasis and detailed views on life after death. The majority claimed having faith in the continuity of the soul, which they did so by affirming reincarnation, the afterlife and the possibility that the soul might travel to another dimension. According to one student, “We all leave this place one day, and we all reincarnate our consciousness in every being”. Furthermore, the Indian students also emphasised the concept of “soul continuity”, which one student explained as follows:

According to me, life is not the one that is alive [sic]. Life is also beyond death. Even if the death of the body has happened, the soul is alive. I believe our body is a vehicle that runs throughout our time on earth, and when the time for the soul to transition arrives, the vehicle is left behind, whereas the soul exists to another realm of reality.

#### 3.2.2. Second Theme: Social Avoidance of the Topic of Death

The second theme concerns the participants’ perceptions regarding how society relates to the topic of death. In particular, the first prevalent thought amongst most of the participants is that death is considered taboo and is a highly avoided and distorted topic. As one Italian student expressed, “I am absolutely convinced that people today shy away from everything related to death and dying. There is no relationship with death; it is simply hidden, and all those situations that remind us of it are avoided”. Similarly, an Indian student shared that talking about death is prohibited because it scares people:

Society as a whole perceives death as taboo. They hesitate to talk about death because they don’t want to think about their loved ones leaving them. People are very scared of death, which is the main reason they avoid talking about it.

Students from both countries expressed that losing someone causes an uncomfortable emotion and, therefore, not talking about it can make people feel better. One Italian student recalled an experience at work, wherein death was regarded as a sad topic: “Death is not talked about, and the proof is that when I talked about it with work colleagues, they all turned their noses up a bit, because [to them] death is a ’sad’ topic”. Another student from India expressed that “Death is a subject that is often avoided by many. It brings out various emotions, the most common being fear. People fear death and the end of their own lives”.

Both the Italian and Indian students perceived that society and its current norms also influenced the way death was perceived. However, the former provided a different perspective. As expressed by one Italian student:

The society in which I live is oriented by the attainment and maintenance of ephemeral standards of beauty, eternal youth, and physical fitness (=health). Therefore, it repudiates any thought that would distract them from all this, much more the thought of death that would put an end to all this.

Such criticism of society’s superficiality is reiterated by six Italian students, one of whom stated, “I am very embittered by the society in which we live, by its total inability to deal with certain issues, belittle them, reduce them to an all concrete and ‘entertainment and distraction’ dimension, only corporeal/aesthetic and extremely empty”.

At the same time, Indian students also made a significant point about death, in that the majority of them focused on societal pressure and expectations following a death in a family. According to one student:

Well, I wouldn’t say it’s not painful to witness the death of your loved one, but the aura and energy that surround you right after an individual’s death are filled with pity, sympathy, and unsolicited advice. Letting people make their journey towards grieving is something that should be allowed. One cannot expect to be all fine and live life just like the day before.

As a result, after witnessing a family experience the death of a loved one, the general public’s negative impressions about the family are formed. One Indian student expressed a related opinion:

In society, death is perceived as a very demotivating and sad concept. They always react to it very negatively and make it feel like everything is about to end … They lose hope and don’t look at the situation in a positive manner to give them strength. The individuals in society would cry, sympathise; some might even state that it is the end of life for the person and there is no hope. So, they end the source of any hope or space for improvement in the individual.

According to the majority of the participants, the COVID-19 pandemic has brought out even more of this superficial societal attitude in dealing with death, catching unprepared people who suddenly find themselves facing different kinds of losses. As one Italian student shared:

COVID-19 had a major impact on me, and I think a little bit on everyone. I personally faced it badly and with fear and dread. I think people are unlikely to get over this past period easily, and we are still carrying on. There has been so much loss, and the fear of losing loved ones has been strong and painful and not at all easy to live with. Over time, I learned to deal with it, but I remembered back in the day how difficult it was and how unprepared the Italian population was.

Similarly, after seeing a fatality during the COVID-19 period, an Indian student shared the following insights:

In the last two difficult years, I have witnessed a lot of deaths. After this experience, I tend to avoid any and all topics of death. As a future psychologist, this would be difficult for me professionally, as I might be unable to aid those experiencing grief or handle any topic surrounding death.

Another Indian student’s account of the second COVID-19 wave expressed similar sentiments: “The second wave of the pandemic was when I experienced anxiousness and grief like no other. It made me feel scared of death and the implications of death in family members”. 

#### 3.2.3. Third Theme: Areas of Use of Death Education

This theme focuses on the three areas of use of death education, which the participants highlighted as being of particular importance. The first area of use concerns the importance of raising awareness of the issue of death was mentioned by 22 students from Italy, about which one student stated:

I think it is important to promote paths inherent in death and mourning, to increase sensitivity to the pain and suffering of others and to recognise and look at them as human beings and not as targets to be done away with.

Similarly, an Indian student stated, “It is important to study death education. The relationship people usually have with death is denial; they usually avoid speaking about it, and they do not do anything to foster a good relationship with it”.

Regarding the importance of raising awareness of the issue of death, it also emerged from the students’ answers that death education interventions are useful both for achieving better self-knowledge and for re-appropriating a topic that has become taboo. As one Italian student reported:

I think that promoting the study of issues of death, bereavement, and palliative care would be very important, which can also help people dig into themselves to find out what they really think, and to remove the taboo of death by making it “public” again but in a healthy way, not only through crime news and violence in movies and video games.

Indian students, meanwhile, emphasised the need to change society’s attitudes towards dying. This is in reaction to the second theme, which identified the issue as a negative societal perception. The students, however, believed that death education could fix this problem. As one student stated:

As we celebrate birth, death should also be celebrated. But it’s easy to say, and it’s hard for someone who goes through it. That’s where the importance of promoting the study of death education comes. Just as we take sex education and related classes, death education should also be provided.

A second area of use for death education concerns the training of professionals working in personal care settings. This was supported by six students from Italy, one of whom shared the following views:

I also believe that it is essential in the fields of medicine, nursing, and psychology to study to acquire death competence, which is necessary to take responsibility for dealing with the person, even before dealing with the patient. The role of these figures is essential from the first meeting with the person who brings a request for help—whether physical and/or psychological—but also at the time of diagnosis, breaking bad news, for the communication of prognosis and possible interventions and for informing [those involved] on the course of the disease, including the sick person and family members. Here, then, the university and specialist pathways should include training in this regard for each respective course of study.

Students from India also placed more emphasis on raising awareness of death so that society’s perceptions could be changed. According to one student, “Promoting studies for issues concerning death, bereavement, and palliative care will definitely foster a better relationship between society and death. It will give some kind of courage to people and help them be prepared”.

As a third area, the importance of the palliative psychologist’s use of death education in accompanying the dying person to the last stage of life is mentioned by six students. As one of the Italian students shared:

Regarding psychological accompaniment to dying, I think it is really inevitable to ensure the presence of a psychologist in that final process of life. I am convinced that this is the most suitable figure to enhance the life lived by the person who is ending his journey and to help him express his experiences and his emotions, but above all, the professional figure capable of authentic and sincere listening.

An Indian student expressed a highly similar sentiment:

I chose this course merely because my uncle was suffering from cancer and because I wanted to help him and my cousins in every way possible. I assumed that learning the course would act as a practical education where I would be able to help my family. I couldn’t be farther from my assumptions, and this course truly helped me handle it in certain ways.

Six participants from Italy also emphasised the use of palliative psychology in supporting the family members of a terminally ill person. As one of them shared:

I discovered the existence of palliative psychologists and their importance in supporting dying persons and their families during this journey, and I find that it is crucial in that it can both help dying persons carry out their last developmental task in a dignified manner and help their families process this grief.

Similarly, one Indian student expressed, “It is important that we address the process of bereavement in times of loss. Once understood and learnt, facilitating the patients’ easy ‘journey’ and ensuring the caretaker or the families’ well-being are made possible”.

In addition, Indian students perceived death education as a potential tool for changing the paradigm of society in handling death. In particular, the students believed that death education can bring a positive attitude in society, increase social support and improve the quality of death amongst dying people. As expressed by one student:

Yes, I believe that society has a very negative notion about death, and courses like palliative and hospice care would help people understand it better. Just as when we are aware that death is certain, and it is still difficult to accept that, this course will help others understand and accept death in a more positive manner.

The majority of Indian students highlighted the lack of such death education courses in India because they had not heard of such services in the country prior to enrolling in this course:

Less importance is given to the concept and to the people fighting death. Hence, there are significantly fewer palliative care centres in the country. The introduction of the course would help individuals fighting with life-and-death situations to gain faith and strength and deal with them without compromising their well-being, which, in turn, will increase the will to fight their illnesses.

#### 3.2.4. Fourth Theme: Impact of the Death Education Course

The fourth and final theme concerns the impact perceived by the students after participating in the death education course. Most of the participants appreciated the opportunity to be able to reflect on and talk about the topic of death during the course, as reported by one Italian student:

I also think that having the opportunity to reflect and talk about death is an important opportunity to become more familiar with the topic and with one’s own emotions and fears, just like what happened to me as a result of attending the course.

Similarly, an Indian student said:

I believe that taking up palliative care has made me open up as a person about the sensitive issues that I used to run away from, mainly death. I can now accommodate my feelings from the perspective of what death means to different people at different times and its essence.

All the students said they felt more aware and knowledgeable about the topic after participating in the course. As expressed by one participant:

Today, I speak about it with a different awareness, certainly more mature, about how true it is that knowledge makes one free. This course helped me confront [the topic] often with my own pain, past and present, and with that of my family members. I learned to understand and not to judge the reactions in the face of pain because no one had taught me how to do so, neither my family nor other people.

An Indian student expressed a similar opinion:

It has indeed influenced my opinion because when you learn palliative psychology, you learn compassion. You understand how terrible the client and their family feel, and that makes you more eager to understand their emotions and thoughts and try to ease their pain. The difference that I see now is that I have a different perspective towards helping people with a terminal illness. Before this, I might have been a little reluctant about this field, but now, after learning about it, I feel how important this course is.

Sixteen participants from Italy claimed to experience talking about death more serenely and naturally. As one student wrote:

The course greatly influenced my opinion on the subject. It has enabled me to gain knowledge and develop an awareness of and serenity in dealing with a subject that I personally used to fear in an anguished way. I now feel able to talk about death with greater peace of mind and spend the skills I have acquired offering psychological support or advocacy to people who may need it.

Along similar lines, Indian students also believed that their negative feelings about dying had changed. As explained by one student:

For me, death was a negative concept—something that I would voluntarily hide from. Even when informed about a death, if near and dead, my emotions either flare up or shut down because I was too scared of the idea of death. Being in this course has helped me in all aspects. I feel more inclined towards helping in providing care and comfort [to those who may need it].

Six students from Italy pointed out that, although they gained more skills and awareness, they also felt the need to deepen their knowledge and maturity on this topic, as one student said:

I believe now, however, that I am not yet fully ready to work in this area, as I still need to fully understand my thoughts and fears with respect to death. However, I do not exclude the possibility of working in this area one day tomorrow. The course helped me understand how important the work can be.

Another student from Italy reported a similar thought and expressed difficulties in dealing with the eventual death of a close loved one, saying, “However, if I were to face the death (of a loved one) myself, I believe I have only acquired the basics of competence. It still has not settled in me the ability to hold the course with maturity and awareness”.

Similarly, Indian students also said that this course increased their competence in dealing with the topic of death, as one student pointed out:

This course, in some sense, has helped me become more comfortable with the topic of dying and not just death as something that happens to everyone. So, yes, I do feel more competent compared to what I was five months ago.

Another consequence of having participated in the course is having a greater interest in the palliative care field as a job possibility. This has been reported by 10 female Italian students, amongst whom one said: “Having acquired some specific skills in palliative psychology has increased my interest regarding working in the field and also awareness of how much in fact the socio-psychological-spiritual part is also involved in this last developmental task”. 

At the same time, Indian students expressed their strong motivation to pursue this profession as a career as a result of joining this course:

The motivation became tremendous through this course. This has made my understanding much broader in terms of the different opportunities and methods to help people in need. Before the course, I was not sure about how the whole process operates, but now that I have completed the course, it is all clear to me. It is an amazing field to work in.

In accordance with the above, an Italian student reported feeling a vocation for such a field of work after participating in the course, saying, “Having participated and taken this course for me was ‘enlightening’. I felt a kind of ‘calling’; in fact, I am highly motivated to continue my growth (theoretical, but also practical) in this speculative and applied field”.

However, some Indian students deferred and expressed that they did not see palliative care as a good career option for them. As one Indian student stated:

Attending the course helped me find structure to the subject. It exposed me to many more concepts, learnings and workings of the field. I definitely do want to contribute to the palliative field; however, I do not see it as a career for myself. Nevertheless, I would love volunteering in the field, as I recognise how my work could aid individuals.

## 4. Discussion

The contribution of this study is that it deepens our understanding of the impacts of a death education and palliative psychology course on the education of undergraduate students enrolled in the master’s programme in psychology in Italy and India, highlighting the similarities and differences between the two different social contexts. As university courses on these topics are rare, the present research wanted to explore whether training in death education and palliative psychology could be perceived as interesting and helpful by groups of students and whether it can facilitate their preparation in the end-of-life field as future psychologists. The results of the present study reveal that, on the one hand, Italian students feel more prepared and confident in dealing with the topic of death after participating in the course. On the other hand, Indian students feel more competent in working in a palliative care context. They also seem more aware of and less preoccupied with issues related to death than the Italian students.

Furthermore, several differences have been found between the two groups of students: the Indian students are older than their Italian counterparts, show higher levels of interest in death and palliative care issues, are more confident with the topic of death and show higher levels of religiosity than Italian students. These results are in accordance with the literature, which states that interest and confidence in the topic of death are positively correlated with age and religiosity [9,19]. Furthermore, higher self-confidence in a professional domain is positively correlated with a higher level of interest in that domain [33]. 

The quantitative results of the study indicate that the Italian students experienced significant changes in their views of death, which they considered more as passing and less as annihilation. According to the literature, this may indicate an improvement in attitudes towards death and in personal well-being, as it has been shown that viewing death as annihilation is positively correlated with depression and negatively correlated with resilience [20,22,24]. 

Both quantitative and qualitative results show that the Italian students feel more knowledgeable and confident in approaching the topic of death following participation in the death education course. This outcome is consistent with past studies, which concluded that most death education courses had had positive impacts in terms of decreased anxiety and fear of death and increased self-confidence in dealing with the topic of death [4,7,8,10,17,22,23,24,34]. 

Similar to this finding, our qualitative data revealed that the Indian students felt more competent to work in a palliative care setting and that the course had raised their awareness and decreased their worries about the concept of death [35,36]. This result highlights the importance of death education in the training of future professionals, such as psychology students, in the field of personal care [37,38].

Furthermore, the qualitative results show that the Italian students, from their initial adherence to their home religion, later chose a more spiritual path. This result is consistent with the literature regarding changes in Italian society from a social structure based on collectively shared religion to an individualisation of beliefs and spiritual dimensions that go beyond conventions [27]. Meanwhile, the Indian students have demonstrated the impact of the country’s cultural and religious framework on their understandings of and beliefs regarding concepts of death and life [39,40]. The connections between death and spiritual development, the transfer to a new life, and the opportunity to grow are consistent with numerous ethnographic studies on the Indian community [41]. The Indian students also perceived this course as a potential instrument for influencing people’s mindsets and bringing positive knowledge to the subject of death. They believed that the social traditions and rituals associated with dying may have had a negative impact [42,43].

Both groups of Indian and Italian students also reported having attitudes that tended towards avoidance of the topic of death. This finding is in agreement with the literature, which shows that in modern society, death is taboo and tends to be collectively removed from daily life because it arouses emotions of distress and anxiety [12,17,20,44,45,46]. 

An additional qualitative finding from the Italian group highlights the importance of employing death education in raising awareness about death and training professionals working in personal care settings. Several international studies have found death education to be effective in increasing self-efficacy and confidence in dealing with the topic of death by reducing associated anxiety among students [4,7,8,9,10,14,15,16,17,22,23,24,47]. Students who will become future health professionals in the field of care complain of a lack or complete absence of training in end-of-life care and, more generally, on the topic of death [7,8,9,16]. In line with international research, integrating death education courses into the education of future nurses, physicians, and psychologists can provide students the opportunity to work on their beliefs, fears, and anxieties associated with death so that they are better prepared and competent in caring for patients [7,8,9,14,15,16]. A significant difference in qualitative responses can be seen from the existing status of palliative care in India and Italy. India stands at a very low level in terms of the quality of death index [48], and coverage for care is limited to cities [49]. The replies from the students are consistent with the earlier surveys, in which students expressed reluctance to work in similar fields due to their limited scope.

However, the death education course alone would not be sufficient; instead, attractive government job opportunities for young professionals must be launched and promoted to encourage young professionals to work in this field [50] 

## 5. Limitations and Future Directions

This study has several limitations. The first limitation is the lack of a control group in each country with which to compare the quantitative results over time. Furthermore, the student samples are small and concern only one university in each country, and some Indian students are unable to complete the questionnaire before and after the course. Thus, the comparison of the quantitative results between the two groups is weaker. Another limitation concerns the structure of the courses, which have strong similarities but are not identically structured. This can be attributed to the fact that it was impossible to change the teaching plans between the two universities. However, given the difficulty in finding university courses that deal with these topics, we tried to make them as similar as possible so that we could conduct the research. Finally, the study is also limited in terms of the lack of responses to the questionnaire from Indian students who have been unable to consistently complete both the pre- and post-course questionnaires.

Future studies could evaluate the impacts of a death education and palliative psychology course in different cultural contexts, use larger samples, include a control group and, finally, structure courses in an identical way.

## Figures and Tables

**Table 1:** Country differences before and after the course.

	Before the Course			After the Course		
	Italy	India	Country Differences	Italy	India	Country Differences
Variables	(N = 63)	(N = 25)	*p*-Value	(N = 51)	(N = 36)	*p*-Value
Demographic variables						
Age	25.44 (7.32)	23.20 (1.66)	0.025	25.71 (8.19)	23.33 (1.94)	0.051
Gender:			0.055			0.051
Female	60 (95%)	20 (80%)		48 (94%)	29 (81%)	
Male	3 (5%)	4 (16%)		3 (6%)	7 (19%)	
Missing	0 (0%)	1 (4%)		0 (0%)	0 (0%)	
Marital status:			0.241			0.213
Single	26 (41%)	16 (64%)		22 (43%)	22 (61%)	
In a relationship	31 (49%)	7 (28%)		25 (49%)	11 (31%)	
Married	5 (8%)	2 (8%)		4 (8%)	3 (8%)	
Missing	1 (2%)	0 (0%)				
Religion:			<0.001			<0.001
Christian	32 (51%)	1 (4%)		27 (53%)	1 (3%)	
Hinduism	0 (0%)	14 (56%)		0 (0%)	18 (50%)	
Other	0 (0%)	7 (28%)		0 (0%)	12 (33%)	
None	31 (49%)	3 (12%)		24 (47%)	5 (14%)	
Religious level	1.71 (0.77)	2.72 (0.89)	<0.001	1.82 (0.87)	2.81 (0.62)	<0.001
Experience variables						
Formal caregiver to end-of-life clients (Yes)	7 (11%)	4 (16%)	0.532	4 (8%)	7 (19%)	0.109
Lost someone close to you in the last 2 years (Yes)	34 (54%)	8 (32%)	0.063	23 (45%)	14 (39%)	0.564
Terminal illness of someone close to you—currently (Yes)	9 (14%)	3 (12%)	0.778	9 (18%)	0 (0%)	0.008
Read something about end-of-life, bereavement and/or palliative care (Yes)	18 (29%)	20 (80%)	<0.001	47 (92%)	30 (83%)	0.204
Master Year:			<0.001			<0.001
1st	47 (75%)	1 (4%)		41 (80%)	0 (0%)	
2nd	16 (25%)	24 (96%)		10 (20%)	36 (100%)	

The values reported in the table are mean (standard deviation) for continuous variables and frequency (percentage) for nominal variables (only Yes for dummy variables). The column “Country differences *p*-value” shows the *p*-values for the Chi-square test for nominal variables and the t-test for continuous variables.

**Table 2:** Regression analysis on target variables before and after the course.

	Target Variables				
Predictor	Interest Total	Confidence Total	Death Is Terminal	Death Is a Passage	Intolerance of Ambiguity
Before the course (N = 88)					
Age	−0.00 (0.01)	0.02 (0.02)	0.04 (0.03)	−0.02 (0.03)	−0.02 (0.02)
Gender (Female = 0, Male = 1)	−0.42 ~ (0.22)	0.03 (0.33)	0.47 (0.52)	−0.75 (0.49)	−0.47 (0.41)
Religious level	−0.12 (0.08)	−0.21 ~ (0.11)	−0.79 *** (0.18)	0.81 *** (0.17)	0.01 (0.14)
Formal caregiver to end-of-life clients (No = 0, Yes = 1)	0.38 ~ (0.20)	0.31 (0.31)	−0.33 (0.48)	0.13 (0.46)	0.25 (0.38)
Lost someone close to you in the last 2 years (No = 0, Yes = 1)	−0.01 (0.13)	−0.04 (0.19)	0.46 (0.30)	0.04 (0.28)	0.22 (0.24)
Terminal illness of someone close to you—currently (No = 0, Yes = 1)	0.03 (0.18)	−0.26 (0.27)	0.43 (0.43)	−0.27 (0.41)	0.67 (0.34)
Read something about end-of-life, bereavement and/or palliative care (No = 0, Yes = 1)	0.38 * (0.17)	0.59 * (0.25)	−0.24 (0.39)	0.07 (0.37)	−0.05 (0.31)
Master year (1st = 1, 2nd = 2)	−0.08 (0.17)	0.43 ~ (0.25)	−0.16 (0.39)	0.21 (0.37)	−0.22 (0.31)
Country (Italy = 0, India = 1)	0.32 (0.21)	1.32 *** (0.32)	1.00 * (0.50)	−0.38 (0.47)	0.02 (0.40)
R-square	25% **	57% ***	29% **	30% ***	12%
After the course (N = 87)					
Age	0.01 (0.01)	0.03 ~ (0.01)	0.03 (0.02)	0.01 (0.02)	0.01 (0.02)
Gender (Female = 0, Male = 1)	−0.20 (0.27)	−0.08 (0.30)	1.32 ** (0.44)	−1.18 ** (0.37)	−0.37 (0.34)
Religious level	−0.03 (0.11)	−0.06 (0.13)	−0.52 ** (0.18)	0.62 *** (0.15)	0.06 (0.14)
Formal caregiver to end-of-life clients (No = 0, Yes = 1)	0.63 * (0.26)	0.44 (0.29)	0.49 (0.42)	−0.27 (0.35)	−0.50 (0.33)
Lost someone close to you in the last 2 years (No = 0, Yes = 1)	0.28 ~ (0.17)	−0.03 (0.19)	0.48 ~ (0.28)	−0.42 ~ (0.23)	−0.11 (0.22)
Terminal illness of someone close to you—currently (No = 0, Yes = 1)	0.04 (0.29)	−0.08 (0.33)	−0.50 (0.48)	0.43 (0.40)	0.03 (0.37)
Read something about end-of-life, bereavement and/or palliative care (No = 0, Yes = 1)	0.24 (0.26)	0.36 (0.30)	−0.28 (0.43)	0.02 (0.36)	−0.70 (0.34)
Master year (1st = 1, 2nd = 2)	0.17 (0.28)	0.08 (0.32)	−0.07 (0.46)	0.17 (0.39)	0.21 (0.36)
Country (Italy = 0, India = 1)	−0.44 (0.33)	0.60 (0.38)	0.88 (0.55)	−0.37 (0.46)	−0.39 (0.43)
R-square	18% ~	20% *	30% ***	31% ***	11%

* The values reported are unstandardised regression coefficients and (standard error). ~ *p* < 0.10; * *p* < 0.05; ** *p* < 0.01; *** *p* < 0.001.

**Table 3:** Course effect on target variables for the Italian sample (N = 35).

Target Variable	Before Course		After Course		Course Effect	
	M	SD	M	SD	t	Cohen’s d
Interest total	4.24	0.58	4.15	0.63	−1.01	−0.17
Confidence total	2.49	0.90	3.16	0.76	4.80 ***	0.81
Death is terminal	3.46	1.40	2.89	1.45	−2.95 **	−0.50
Death is a passage	2.91	1.31	3.40	1.31	3.68 **	0.62
Intolerance of ambiguity	3.57	0.95	3.63	0.82	0.41	0.07

* ~ *p* < 0.10; * *p* < 0.05; ** *p* < 0.01; *** *p* < 0.001.

## Data Availability

The data that support the findings of this study are openly available in Research Data Unipd at 10.25430/researchdata.cab.unipd.it.00000775 (accessed on 29 November 2022).
